# Validation of the Asia-Pacific colorectal screening score and its modified versions in predicting colorectal advanced neoplasia in Chinese population

**DOI:** 10.1186/s12885-022-10047-y

**Published:** 2022-09-07

**Authors:** Yunxin Kong, Lin Zhuo, Dong Dong, Lang Zhuo, Peian Lou, Ting Cai, Siting Chen, Jianqiang Pan, Yihuan Gao, Hang Lu, Yue Ma, Zongmei Dong, Xiaohu Luo, Hongying Zhao

**Affiliations:** 1 Cancer Prevention office, Xuzhou Cancer Hospital, Huancheng Road 131, Gulou District, Xuzhou, 221000 Jiangsu China; 2 Endocrinology and metabolism, Tianjin Medical University General Hospital, Tianjin, 300000 China; 3 School of Public Health, Xuzhou Medical University, Tong Shan Road 209, Yunlong District, Xuzhou, 221004 Jiangsu China; 4 Department of Control and Prevention of Chronic Non-communicable Diseases, Xuzhou Center for Disease Control and Prevention, Xuzhou, 221004 China; 5 School of Management, Xuzhou Medical University, Xuzhou, Jiangsu Province, 221004 China; 6 Toxicology Lab, Xuzhou Cancer Hospital, Xuzhou, 221000 China; 7 Department of Medical Oncology, Xuzhou Cancer Hospital, Xuzhou, 221000 China

**Keywords:** Colorectal neoplasia, Mass screening, Early detection of cancer, Risk assessment, Validation studies as topic

## Abstract

**Background:**

Colorectal cancer is one of the most common cancers in the world. Several studies suggest using the Asia-Pacific colorectal screening (APCS) score and its modified versions to select high-risk populations for early colonoscopy, but external validation remains rare, and which score should be selected for CRC screening in China is unclear. Validation of multiple scores in the same population might help to choose the best performing score.

**Methods:**

We conducted a cross-sectional study under the framework of Cancer Screening Program in Urban China, data from asymptomatic colorectal cancer screening in Xuzhou was used to validate the APCS score, the colorectal neoplasia predict (CNP) score, the Korean colorectal screening (KCS) score, the Modified APCS score and the 8-point risk score in predicting colorectal advanced neoplasia (CAN).

**Results:**

1804 subjects were included in the analysis and 112 CAN (6.21%) was detected. In each score, the detection rate of CAN was higher in the high-risk group than in the non-high-risk group (*P* < 0.05), and the *RR* (95%*C.I.*) ranged 2.20 (1.50–3.22) [8-point risk] to 4.00 (2.41–6.65) [Modified APCS]. The c-statistics (95%*C.I.*) of the scoring systems ranged from 0.58 (0.53–0.62) [8-point risk] to 0.65 (0.61–0.69) [KCS]. The sensitivity (95%*C.I.*) of these systems ranged from 31.25 (22.83–40.70) [8-point risk] to 84.82 (76.81–90.90) [Modified APCS], while the specificity (95%*C.I.*) ranged from 43.50 (41.12–45.90) [Modified APCS] to 83.81 (81.96–85.53) [8-point risk]. Using the APCS scoring system as a comparator, the net reclassification improvement (NRI) of each modified version ranged from − 10.34% (95%*C.I.*: − 22.63 to 1.95%) [8-point risk] to 4.79% (95%*C.I.*: − 1.50% to 11.08) [KCS]. The colonoscopy resource load (95%*C.I.*) ranged from 9 [1–3] [8-point risk] to 11 [3–5] [APCS and Modified APCS].

**Conclusions:**

The APCS score and its modified versions have certain ability to predict the risk of advanced neoplasia and reduce the resource load. The modified APCS score and the KCS score seemed the preferable systems to classify high risk subjects based on its high *RR*, sensitivity and predictive ability in the selected population. Future research could focus on adding risk factors or combining with laboratory test results to improve the predictive power of the scoring system.

## Introduction

Colorectal cancer is one of the most common cancers in the world, more than 1.9 million new colorectal cancer cases and 935,000 deaths were estimated to occur in 2020, representing about one in 10 cancer cases and deaths [6]. In China, colorectal cancer is also one of the commonly diagnosed cancers. A recent study shown that colorectal cancer ranks second in incidence and fourth in mortality, with 408,000 cases and 196,000 deaths, remains a major public health problem [7].

Screening and early intervention have been clearly demonstrated to be effective in improving survival and preventing the occurrence of colorectal cancer [8, 9]. Colonoscopy is regarded as the gold standard. In October 2012, the government of China initiated the population-based Cancer Screening Program in Urban China (CanSPUC), which targeted five types of cancer that are most prevalent in urban areas, including CRC. The CanSPUC now covers 29 provinces in China. Eligible participants aged 40–74 years old are recruited in the communities of the study regions and invited to undertake cancer screening free of charge. Participants are first invited to take a cancer risk assessment, and those who are evaluated to be high risk for specific types of cancer are recommended to take appropriate screening intervention per study protocol. For CRC screening, participants who met the high-risk conditions for CRC are recommended to take colonoscopy at tertiary-level hospitals designated by the programme. However, colonoscopy resources in Asian country like China are relatively limited, and due to some reasons such as pain, complicated preparation, the participation rate of colonoscopy screening in CanSPUC is not as good as expected (only 14%) [10].

To optimize efficiency of resources, the updated Asia Pacific Consensus Recommendations on colorectal cancer screening [11] recommend using Asia-Pacific Colorectal Screening (APCS) score [12] to select high-risk patients for colonoscopy. The APCS score was based on the risk factors identified in Asian populations above 50 years of age from 17 centers in 11 Asian cities, aimed to stratify risk for colorectal advanced neoplasia (CAN) in asymptomatic Asian subjects. Since it include only sex, age, family history and smoking habits without including obesity, diabetes and other possible risk factors, there is opportunities for further improvement on the predictive value of the scoring system [11]. Research team from the Chinese University of Hong Kong developed the Colorectal neoplasia predict (CNP) score [1] and the Modified APCS score [2] by recruiting Chinese asymptomatic screening participants undergoing a colonoscopy in Hong Kong from 2008 to 2012. In the modified versions, Body Mass Index (BMI) and diabetes were added as risk factors and all risk factors were re-assigned. Research teams in South Korea and Japan have also developed the Korean Colorectal Screening (KCS) score [3] and the 8-point risk score [4] based on the APCS score. However, external validation of these risk scoring systems remains rare, and which score should be selected for colorectal cancer screening in China is unclear.

A systematic review and meta-analysis suggest that validation of multiple scores in the same population might help to choose the best performing score for a given study population [5]. Xuzhou is the central city of the Huaihai Economic Zone (which has a population of 119 million, covers an area of 178,000 km^2^ and consists of 20 cities), located at the junction area of four provinces (Jiangsu, Anhui, Shandong and Henan), southeast of the North China Plain, gateway to East China. This study conducted a cross-sectional study under the framework of Cancer Screening Program in Urban China (CanSPUC), data from asymptomatic colorectal cancer screening in Xuzhou was used to validate the APCS score and its modified versions in predicting CAN and provided reference for the selection of colorectal cancer screening tools in China.

## Methods

### Study population

We conducted a cross-sectional study under the framework of Cancer Screening Program in Urban China (CanSPUC). CanSPUC is an ongoing national cancer screening program in urban areas of China, and Xuzhou joined the program in August 2014. Briefly, a cluster sampling method was adopted to conduct simple random sampling with the community as a group in the main urban area of Xuzhou. Residents living in selected communities aged 40–74 years old were approached by trained staff by means of phone calls and personal encounter. After obtaining signed written informed consent, all the eligible participants were interviewed by trained staffs to collect information about their exposure to risk factors and to evaluate their cancer risk using conditions set by the National Cancer Center. To optimize use of the limited colonoscopy resources and to enhance the detection rate of colorectal neoplasia, only participants who met the high-risk conditions for colorectal cancer were recommended to undergo colonoscopy examination at Xuzhou Cancer Hospital designated by the programmer free of charge.

For the present analyses, we used the data of the colorectal cancer screening between August 2014 and August 2021 in Xuzhou. Inclusion and exclusion criteria were used and subjects who met the following conditions were included in the study:

- age ranged 50–74 years old,
- informed consent form was signed,
- risk assessment questionnaire was completed (demographic and socioeconomic statuses, self-reported medical history and lifestyle characteristics were collected),
- colonoscopy screening was completed in the designated hospital (Xuzhou Cancer Hospital),
- no history of colorectal cancer, colorectal adenoma, or colorectal polyp,
- no colorectal cancer related treatment or colorectal resection before screening.

From August 2014 to August 2021, 116,047 participants completed the questionnaire, of which 12,496 (10.77%) met the high-risk conditions for colorectal cancer and were recommended to undergo colonoscopy. Of the 3264 (26.12%) participants who completed colonoscopies, 1804 eligible subjects were included in the analysis.

This study was approved by the Ethics Committee of Xuzhou Cancer Hospital (approved number: 2018-02-23-H01).

### Colonoscopy screening

The nature, benefits and risks of colonoscopy were explained to all subjects prior to the examination and the colonoscopy risk notification form signed. We used polyethylene glycol (HYGECON^R^, Jiangxi Hygecon Pharmaceutical Co., Ltd., China) as a standard bowel preparation regime for all participant, an electrocardiogram was also performed before colonoscopy to prevent unexpected events. A team of experienced physicians and colorectal surgeons performed all colonoscopy procedures at the endoscopy Center of Xuzhou Cancer Hospital. All abnormal findings were pathologically examined in accordance with clinical procedures, and the results and images were uploaded to the project information system. CAN were recorded as positive and other conditions as negative. CAN was defined as colorectal cancer or any colorectal adenoma which measuring 1 cm or more in diameter, or high-grade dysplasia, or tubular-villous histologic features. In order to ensure the quality of the examination, the quality control team composed of the chief physician and the deputy chief physician reviewed all the results.

### Risk stratification

The APCS score and its modified versions were used to stratify the risk of CAN in eligible subjects. A total of 5 existing scoring systems were included in the study, Table 1 summarizes the population, key feature, predictor variables, computational algorithm and prediction effect of each scoring system according to the TRIPOD Statement’s checklist [13]. For the CNP score and the Modified APCS score, age scores of subjects aged 71 to 74 in this study were referenced to ≥70 years old.

**Table 1:** Existing scoring systems for risk prediction of colorectal advanced neoplasia

Scoring systems	Investigators	Population	Outcome	Scoring algorithm	High risk criteria	C-statistic
Asia-Pacific Colorectal Screening (APCS) score	Yeho et al. (2011) [12]	Derivation cohort: 860 subjects from 11 Asian cities Validation cohort: 1892 subjects from 11 Asian cities	CAN	Age (< 50: 0; 50–69: 2; ≥70: 3)	≥4 (Max. = 7)	Derivation cohort: 0.66 (0.62–0.70) Validation cohort: 0.64 (0.60–0.68)
				Sex (male: 1; female: 0)		
				Family history for first-degree relationship (yes: 2; no: 0)		
				Smoking (yes: 1; no: 0)		
Colorectal neoplasia predict (CNP) score	Wong et al. (2013) [1]	Derivation cohort: 2000 subjects from Hong Kong Validation cohort: 3220 subjects from Hong Kong	CN	Age (50–55: 0; 56–70: 1)	≥3 (Max. = 6)	Derivation cohort: 0.62 (0.61–0.63) Validation cohort: 0.62 (0.61–0.63)
				Sex (male: 1; female: 0)		
				Family history for first-degree relationship (yes: 1; no: 0)		
				Smoking (yes: 1; no: 0)		
				BMI (< 25 kg/m^2^: 0; ≥25 kg/m^2^: 1)		
				Diabetes (yes: 1; no: 0)		
Korean Colorectal Screening (KCS) score	Kim et al. (2014) [3]	Derivation cohort: 3561 subjects from Korean Validation cohort: 1316 subjects from Korean	CAN	Age (< 50: 0; 50–69: 2; ≥70: 4)	≥4 (Max. = 8)	Validation cohort: 0.68 (0.61–0.76)
				Sex (male: 1; female: 0)		
				Family history for first-degree relationship (yes: 1; no: 0)		
				Smoking (yes: 1; no: 0)		
				BMI (< 25 kg/m^2^: 0; ≥25 kg/m^2^: 1)		
Modified APCS score	Sung et al. (2017) [2]	Derivation cohort: 3829 subjects from Hong Kong Validation cohort: 1915 subjects from Hong Kong	CAN	Age (50–54: 0; 55–64: 1; 65–70: 2)	≥3 (Max. = 6)	Validation cohort: 0.65 (0.61–0.69)
				Sex (male: 1; female: 0)		
				Family history for first-degree relationship (yes: 1; no: 0)		
				Smoking (yes: 1; no: 0)		
				BMI (< 23 kg/m^2^: 0; ≥23 kg/m^2^: 1)		
8-point risk score	Sekiguchi et al. (2018) [4]	Derivation cohort: 5218 subjects from Japan	CAN	Age (40–49: 0; 50–59: 2; 60–69: 3; ≥70: 3.5)	≥5 (Max. = 8)	Derivation cohort: 0.70 (0.67–0.73) Internal validation: 0.70 (0.67–0.73)
				Sex (male: 1; female: 0)		
				Family history for first-degree relationship (presence of ≥2 first-degree relatives with colorectal cancer: 2; others: 0)		
				Smoking (≤18.5 pack-years: 0; > 18.5 pack-years: 1)		
				BMI (≤22.5 kg/m^2^: 0; > 22.5 kg/m^2^: 0.5)		

*CAN* Colorectal advanced neoplasm, *CN* Colorectal neoplasm

### Statistical analysis

Statistical analysis was performed with Stata 16.0. A two-tailed *P* value of < 0.05 was considered statistically significant. The Pearson Chi-square test and relative risk (*RR*) was used to compare the detection rate of CAN in the high-risk and non-high-risk groups classified by each score. The sensitivity, the specificity, the positive predictive value (PPV) and the negative predictive value (NPV) were used to evaluate the accuracy of the predictive strategy. The c-statistics was used to measure the discriminatory power between those with and without CAN. The net reclassification improvement (NRI) was used to compare the prediction ability of the modified versions with the APCS score. The NRI is an index to compare the prediction accuracy of two models and measure for evaluating improvements in risk predictions. It amalgamates information found in reclassification tables into a single value, meaning that the NRI contains information about both the number of individuals whose classification changed from incorrect to correct with the new prediction model and the number of individuals whose classification changed from correct to incorrect.

## Results

### Characteristics of participants

**Fig. 1:**
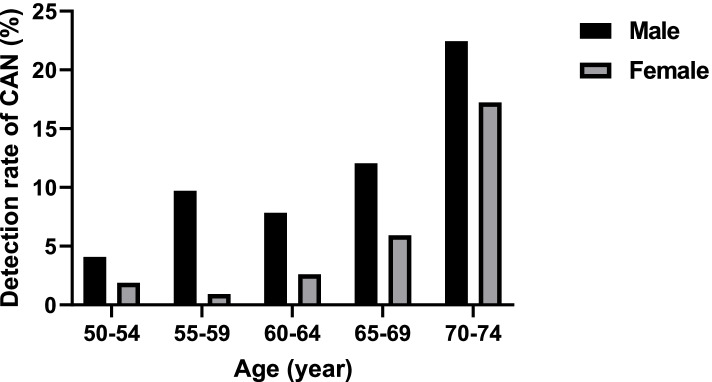
Detection rate of CAN by sex and age. CAN: colorectal advanced neoplasm

A total of 1804 subjects were included in the analysis and the average age (SD) was 59.98 (6.13) years, male accounted for 50.44% (910/1804) (Table 2). 784 subjects (43.36%) had a history of smoking, and most people (78.11%) had a BMI of 23 kg/m^2^ or greater. Only a few people (9.98%) had diabetes, and 15.24% of the subject had a family history of colorectal cancer in first-degree relatives. In the individuals included in the analysis, 112 CAN (6.21%) was detected, including 9 colorectal cancer and 103 advanced adenomas. The detection rate of CAN by sex and age is shown in Fig. 1.

**Table 2:** Characteristics of individuals included in the analysis (*N* = 1804)

Characteristics	N (%)	Characteristics	N (%)
**Age (years)**		**Cigarette smoking (Current or past)**	
50–54	436 (24.17)	No	1020 (56.54)
55–59	434 (24.06)	Yes	784 (43.46)
60–64	472 (26.16)	**BMI (kg/m**^**2**^**)**	
65–69	376 (20.84)	< 23	395 (21.89)
70–74	86 (4.77)	23–25	498 (27.61)
**Sex**		≥25	911 (50.50)
Male	910 (50.44)	**Diabetes**	
Female	894 (49.56)	No	1624 (90.02)
**Family history of colorectal cancer (first degree relatives)**		Yes	180 (9.98)
No	1529 (84.76)		
Yes	275 (15.24)

### Risk stratification results

The high-risk rate (95%*C.I.*) of the APCS score, the CNP score, the KCS score, the Modified APCS score and the 8-point risk score were 49.39% (47.06–51.72%), 48.12% (45.79–50.45%), 53.82% (51.49–56.15%), 58.26% (55.94–60.55%) and 17.13% (15.42–18.95%), respectively. The Modified APCS score is the highest and the 8-point risk score is the lowest. The detection rate (95%*C.I.*) of CAN in high-risk groups of each score were 9.20% (7.39–11.29%), 9.33 (7.48–11.47%), 9.47% (7.71–11.49%), 9.04%(7.37–10.94%) and 11.33% (8.02–15.40%), respectively. In each score, the detection rate of advanced adenoma was higher in the high-risk group than in the non-high-risk group (all *P* < 0.05), and the *RR* (95%*C.I.*) ranged 2.20 (1.50–3.22) [8-point risk] to 4.00 (2.41–6.65) [Modified APCS] (Table 3).

**Table 3:** Risk stratification results and CAN detection according to each scoring system

Scoring systems	High risk		Non-high-risk		***RR*** (95%***C.I.***)	***P***
	N (%)	CAN (%)	N (%)	CAN (%)		
APCS	891 (49.39)	82 (9.20)	913 (50.61)	30 (3.29)	2.80 (1.86–4.21)	< 0.001
CNS	868 (48.12)	81 (9.33)	936 (51.88)	31 (3.31)	2.82 (1.88–4.22)	< 0.001
KCS	971 (53.82)	92 (9.47)	833 (46.18)	20 (2.40)	3.95 (2.46–6.34)	< 0.001
Modified APCS	1051 (58.26)	95 (9.04)	753 (41.74)	17 (2.26)	4.00 (2.41–6.65)	< 0.001
8-point risk	309 (17.13)	35 (11.33)	1495 (82.87)	77 (5.15)	2.20 (1.50–3.22)	< 0.001

*RR* Relative risk, *CI* Confidence interval, *CAN* Colorectal advanced neoplasm

### Performance characteristics

The c-statistics (95%*C.I.*) of the scoring systems ranged from 0.58 (0.53–0.62) [8-point risk] to 0.65 (0.61–0.69) [KCS]. The sensitivity (95%*C.I.*) of these systems ranged from 31.25 (22.83–40.70) [8-point risk] to 84.82 (76.81–90.90) [Modified APCS], while the specificity (95%*C.I.*) ranged from 43.50 (41.12–45.90) [Modified APCS] to 83.81 (81.96–85.53) [8-point risk] (Table 4).

**Table 4:** Performance characteristics of each scoring systems

Scoring systems	c-statistics (95% ***C.I.***)	Sensitivity (95% ***C.I.***)	Specificity (95% ***C.I.***)	PPV (95% ***C.I.***)	NPV (95% ***C.I.***)
APCS	0.63 (0.58–0.67)	73.21 (64.02–81.14)	52.19 (49.78–54.59)	9.20 (7.39–11.29)	96.71 (95.34–97.77)
CNS	0.63 (0.59–0.67)	72.32 (63.07–80.36)	53.49 (51.08–55.89)	9.33 (7.48–11.47)	96.69 (95.33–97.74)
KCS	0.65 (0.61–0.69)	82.14 (73.78–88.74)	48.05 (45.64–50.46)	9.47 (7.71–11.49)	97.60 (96.32–98.53)
Modified APCS	0.64 (0.61–0.68)	84.82 (76.81–90.90)	43.50 (41.12–45.90)	9.04 (7.37–10.94)	97.74 (96.41–98.68)
8-point risk	0.58 (0.53–0.62)	31.25 (22.83–40.70)	83.81 (81.96–85.53)	11.33 (8.02–15.40)	94.85 (93.60–95.91)

*PPV* positive predictive value, *NPV* Negative predictive value, *CI* Confidence interval

### Reclassification performances

Using the APCS scoring system as a comparator, the NRI of the CNP score (0.41, 95%*C.I.*: − 7.06 to 7.88%) was statistically similar (*P* = 0.915). Considering the small study population, to avoid overreliance on *p*-values, although *P* > 0.05, the statistical accuracy of the KCS score (NRI: 4.79, 95%*C.I.*: − 1.50% to 11.08) and the Modified APCS score (NRI: 2.92, 95%*C.I.*: − 5.00 to 10.84%) was considered better than that of the APCS score, the statistical accuracy of the 8-point risk score (NRI: -10.34, 95%*C.I.*: − 22.63 to 1.95%) was considered lower than that of the APCS score (Table 5).

**Table 5:** The Reclassification performances of each risk scoring system

Scoring systems	Risk stratification	APCS		Reclassified (%)	NRI (95%***C.I.***)	***P***
		High risk	Non-high-risk			
**CNS**						
CAN	High risk	73	8	9.88	0.41%	0.915
	Non-high-risk	9	22	29.03	(−7.06 to 7.88%)	
Others	High risk	655	132	16.77		
	Non-high-risk	154	751	17.02		
**KCS**						
CAN	High risk	81	11	11.96	4.79%	0.136
	Non-high-risk	1	19	5.00	(−1.50% to 11.08)	
Others	High risk	738	141	16.04		
	Non-high-risk	71	742	8.73		
**Modified APCS**						
CAN	High risk	79	16	16.82	2.92%	0.470
	Non-high-risk	3	14	17.65	(−5.00 to 10.84%)	
Others	High risk	710	246	25.73		
	Non-high-risk	99	637	13.45		
**8-point risk**						
CAN	High risk	35	0	0	-10.34%	1.901
	Non-high-risk	47	30	61.04	(−22.63 to 1.95%)	
Others	High risk	274	0	0		
	Non-high-risk	535	883	37.73		

*NRI* Net reclassification improvement, *CI* Confidence interval, *CAN* Colorectal advanced neoplasm

### Resource load

The number of individuals needed to screen and undergo colonoscopy to detected one CAN using the APCS score, the CNP score, the KCS score, the Modified APCS score and the 8-point risk score were 11 (95%*C.I.*: 10–12), 11 (95%*C.I.*: 10–11), 11(95%*C.I.*: 10–11), 11 (95%*C.I.*: 10–12) and 9 (95%*C.I.*: 8–10). All scoring systems reduce the resource load compared to not using them (16, 95%*C.I.*: 15–17), and the 8-point risk score having the greatest reduction.

## Discussion

This cross-sectional study validated the performance of the APCS score and its modified versions in an asymptomatic population in China. The results show that all scoring systems have certain ability to predict the risk of CAN and reduce the resource load. The modified APCS score and the KCS score seemed the preferable systems to classify high risk subjects based on its highest *RR*, sensitivity and predictive ability in the selected population.

The most important finding of this study is that the APCS score and its modified versions have certain ability to predict CAN in asymptomatic population in Xuzhou. This result is consistent with previous validation of the APCS score in Beijing [14] and Ningxia [15], which may mean that risk scoring scores can be used as a preliminary screening for colorectal cancer screening in China. Even more, as the updated Asia Pacific Consensus Recommendations on colorectal cancer screening recommended [11], the risk scoring system can select high-risk patients for colonoscopy and reduce the colonoscopy resource load required to detect one CAN. Since questionnaire survey is one of the basic methods of colorectal cancer screening in China, the use of the scoring system as a preliminary screening may improve the cost-effectiveness of colorectal cancer screening.

Although the same variables are present in each model and there are only minor differences in c-statistics, PPVs and NPVs, with the change of variable assignments and cut-offs, the sensitivity and specificity of each model for CAN detection were different. Compared with the APCS score, the CNP scores performed similarly. The modified APCS score and the KCS score improved the sensitivity and reduced the missed diagnosis of CAN, but the specificity decreased and the misdiagnosis increased. On the contrary, the 8-point risk score improved the specificity and reduced the misdiagnosis, but the sensitivity was decreased, which was easy to cause missed diagnosis. Since colorectal cancer screening is the process of detecting and intervening early-stage colorectal cancers and precancerous lesions in asymptomatic population [16–18], it is more important to reduce missed diagnoses with little difference in resource loads. The modified APCS score and the KCS score seemed to be preferable systems to classify high risk subjects based on its highest sensitivity in the selected population. They are also modified versions with improved diagnostic accuracy compared to the APCS score. However, it is important to note that established scoring systems was used in this external validation, and the comparison of different scoring systems was based on the identified cut-off points. In the practical application of risk scoring models, the cut-off points may need to be flexibly changed according to the colonoscopy resources, and the diagnostic performance of the model for CAN will change with the change of cut-off point. Since a too high cut-off point in the risk score model will cause more missed diagnosis, while a too low cut-off point will cause more misdiagnosis, the value of the cut point need to be balanced.

It is also important to note that the population on which the models are tested is not a true average-risk population. To optimize the use of a limited resource and increase prevalence of CAN, only participants who met the high-risk conditions for colorectal cancer were recommended to undergo colonoscopy examination when CanSPUC was conducted [10]. This leads to a higher high-risk rate in the risk stratification results of this study. At the same time, since most of the excluded people who did not meet the CanSPUC’s high-risk conditions for CRC may not be exposed to or less exposed to the high-risk factors in the scoring systems and would be assigned to the non-high risk group, the CAN detection rate of these people is also more likely to be lower than that of the “non-high risk subjects” in this study, the effect of the scoring models in this study might be underestimated. The report of the TARGET-C [19, 20] may support this inferences. In the risk-adapted screening group of TARGET-C, based on the modified APCS score, high-risk subjects (18.9%) were referred for colonoscopy and low-risk ones (80.7%) were referred for FIT. The detection rate of CAN was 5.30% (78/1472) in high-risk individuals and 0.81% (51/6279) in low-risk individuals [20].

Using the APCS scoring system as a comparator, the modified APCS score, the KCS score and the 8-point risk score added BMI as a risk factor, while the CNP score added BMI and diabetes as risk factors. BMI is a typical value derived from the weight and height to define overweight (25 ≤ BMI < 30) and obesity (BMI ≥ 30) in adult. Obesity is regarded as one of the key risk actors for the pathogenesis of colorectal cancer with 11% of colorectal cancer cases in Europe linked to being overweight [21, 22]. According to the 2018 WCRF/AICR report [23], each 5 kg/m^2^ increase in BMI was associated with a 5% increase in colorectal cancer risk (*RR* = 1.05, 95%*C.I.*: 1.03 to 1.07). Diabetes mellitus is also widely believed to be involved in the development of colorectal cancer. In a pooled analysis of 19 prospective population-based cohorts in East and South Asia, Chen et al. [24] found a 41% increased risk of colorectal cancer in patients with diabetes (*HR* = 1.41, 95%*C.I.*: 1.26–1.57). The China Kadoorie Biobank (CKB) study, which included a follow-up study of 500,000 participant, found a 44% increased risk of colorectal cancer among screen-detected diabetics (*HR* = 1.44, 95%*C.I.*: 1.18–1.77) [25]. In addition, several factors considered to be associated with colorectal cancer were not added to the scoring system. Ulcerative colitis [26], red and processed meat intake [23, 27] and excessive alcohol consumption [23, 28] are considered risk factors for colorectal cancer. Regularly aspirin taking [29, 30], dietary fiber intake [23, 31] and reasonable physical activity [23, 32] have been shown to reduce the risk of colorectal cancer. Further improvements of the scoring system by continuing to add risk factors may improve the predictive power of CAN, and which factors need be added need to be explored in future research.

To identify high-risk individuals of colorectal cancer accurately, several recent studies had attempted to combine risk-scoring systems with laboratory test results [20, 33, 34]. Since FIT is the most widely used stool-based test and has convenience, speed and economic advantages, it is of course the first choice for the combination of risk scoring system. Chen et al. [20] conducted a randomized controlled trial in Chinese population, and suggested that the individualized screening strategy combining the modified APCS score and FIT could ensure a higher screening participation rate, and the detection rate of CAN was higher than that of FIT alone. Sekiguchi et al. [33] combined the 8-point risk score with FIT and found that the sensitivity of CAN diagnosis was improved compared with that of FIT alone. Park et al. [34] used fecal hemoglobin (f-Hb) concentration as one of the risk factors to establish a scoring system for CAN, with a c-statistic of 0.75(0.73–0.78). The combined application of risk scoring system and laboratory test results may become the development trend of colorectal cancer screening.

This study has several strengths. First, to our knowledge, this is the first cross-sectional study that validated the performance of the APCS score and its modified versions in asymptomatic population in China. Second, this study was conducted under the framework of CanSPUC, which used rigorous standards to guarantee the integrity and accuracy of the collected data, including a review mechanism to ensure the quality of data and development of a data system to monitor all the processes of the study. Thirdly, we used several evaluation indices to evaluate the prediction ability of the scoring systems from various aspects.

This study also has several limitations. First, for practical reasons, only the colorectal cancer screening data of asymptomatic population in Xuzhou were used in this study. Second, although cluster sampling was used during the population recruitment, participation in questionnaire and colonoscopy was voluntary, which may lead to selection bias. Moreover, The sample size is limited, given the low prevalence of CAN even in the setting of higher-than-average risk participants. This is especially true for the number of colorectal cancers, which is the most important outcome to detect.

In summary, in this external validation, the APCS score and its modified versions have certain ability to predict the risk of advanced neoplasia and reduce the resource load. The modified APCS score and the KCS score seemed the preferable systems to classify high risk subjects based on its high *RR*, sensitivity and predictive ability in the selected population. Future research could focus on adding risk factors or combining with laboratory test results to improve the predictive power of the scoring system.

## Data Availability

The data generated in this study are available upon request from the corresponding author.
